# Supplementary material for: Career orientations of medical students: A Q-methodology study
Source: PLoS One. 2021 May 21;16(5):e0249092. doi: 10.1371/journal.pone.0249092 (PMC8139450; doi:10.1371/journal.pone.0249092)
Supplement: S1 Appendix — (DOCX) [file pone.0249092.s001.docx]

S1 Appendix.

Questionnaire

| **#** | **Question** | **Answer** |
| --- | --- | --- |
| 1. | What is your gender? | Female; Male; Other |
| 2. | What is your age? | Numeric |
| 3. | What is your city and country of birth? | Open answer |
| 4. | What is the country of birth of your parents? | Both in the Netherlands; Father in the Netherlands, mother elsewhere; Mother in the Netherlands, father elsewhere; Both not born in the Netherlands. |
| 4a. | What is the country of birth of your mother/father? | Open answer |
| 5. | Did at least one of your parents graduated at an applied university or university? | Yes; No |
| 6. | In which year of medical school are you? | Numeric |
| 7. | Are you planning on following a medical specialty after medical school? | Yes; No; I don’t know; Other, please specify |
| 7a. | If not, why not? | Preference for a profession where no specialisation is needed; No interest in working as a medical specialist; No interest in working as a physician; Other, please specify. |
| 8. | If today was the day you finished medical school, which specialty would you choose today? If you don’t want to pursue a medical specialty, what is it you would like to do? | List of 38 specialties; don’t know/no preference (yet); other, please specify |
| 9. | Did your specialty of preference change in the past 12 months? | Yes; No |
| 10. | If so, what was the specialty of your previous preference? | Open answer |
| 11. | If so, what made you change your mind? | Open answer |
| 12. | Can you sort these aspects from very important (1) to least important (7). You can use every number once. | Life style factors;  Salary and prestige;  Intellectual challenging work;  Career perspectives;  Role models within specialties;  Financial security and job security;  Medical content. |
