# Supplementary material for: Career orientations of medical students: A Q-methodology study
Source: PLoS One. 2021 May 21;16(5):e0249092. doi: 10.1371/journal.pone.0249092 (PMC8139450; doi:10.1371/journal.pone.0249092)
Supplement: Attachment — Submitted filename: Article Review _10.22.2020.docx [file pone.0249092.s002.docx]

Peer-review - Career orientations of medical students: A Q-methodology study

**Summary of research and overall impression**

The authors set out to understand the actual decision-making process medical students go through when choosing a specialty. The research aim and analytical approach are clearly stated as presented by the question of understanding the process of medical specialty preference formation and the utilization of Q-methodology. This hybrid methodology included qualitative and quantitative methods ensuring results could be presented in data form as well as presenting anonymized excerpts of participant voices to further support the quantitative findings. This study contributes to and advances the existing literature on specialty choice of medical students. This is a relevant and on-going topic that needs continued investigation and exploration.

The following is a breakdown of various sections of the study with compliments and comments related to clarification and technical structure.

**Mismatch**

The mismatch of supply of medical professionals and societal healthcare demands was approached by understanding the career aspirations of medical students. Specifically, addressing the actual decision-making process students go through when choosing a specialty. There is a grammatical error in line 41.

**Career aspirations**

This section presents a good vetting of the literature related to the understanding of medical specialty choice and does a good job of honing in on the decision-making process as the focal point for this study. There are grammar and punctuation errors in lines 81, 83 and 91.

**Research aim**

The research aim and analytical approach are clearly stated as presented by the question of understanding the process of medical specialty preference formation and the utilization of Q-methodology. There are grammar and punctuation errors in lines 102 and 103.

**Methods**

Study design, Context and Participants

The study design, context and participants sections are each well-written. There are grammar and punctuation errors in lines 120, 121.

Statement set

The process by which the Statement set was developed is thorough and seemingly well-vetted. The authors are serious and invested in the design of the Q-sort as is evidenced by their meticulous vetting process of reviewing the literature related to specialty preference, engaging clinical and medical education professionals in the design of the statement set, piloting the statement set among students, and revising the statements with feedback obtained to initiate the official study. There are grammar and punctuation errors in lines 146 and 149.

Data Collection

The description of the sorting process needs clarity and more detail in the description. The authors may want to consider using a series of visuals to depict each phase of the sorting process along with a more detailed description of each phase of the sorting process that ultimately leads to the final product in Figure 1. Since data is vital to any study, it is important to also include, perhaps as an addendum a list of the demographic questions on the paper-based questionnaire. Including these details will show transparency and enhance the validity of your overall study.

Analysis

The analysis, as it is framed, supports the study. For clarity on the idealized rankings, it would be helpful to include in the analysis, the statements that support each pattern.

Ethical Considerations

This section is well-outlined.

**Results**

The introductory paragraph of this section initiates a good summary and would be more strongly supported by explicitly stating the three main findings: life-long learning, work-life balance, and career achievement respectively. I suggest considering modification of the construct of the introductory paragraph to include these as it frames the detailed explanation you provide for each of the findings throughout the remainder of this section.

For each of the career orientations you have identified, you attempt to address their validity with your hybrid q-data. The information you present is supportive and might better serve the reader with consistent organization and more definitive grouping of the results as important, unimportant and neutral. I share the following for career orientation 1 as an example and follow with a note for career orientation 2:

Career orientation 1: Lifelong learning as a calling

It is helpful that you unpack the rankings and validity of the statements on your Q-sort that support this career orientation. As you reference Table 1, you should indicate which pattern (1, 2 or 3) to which this section relates. I question if you are attempting to take the results of this portion of the Q-sort and further organize the results into most important, neutral and unimportant? If so, it is best to explicitly state that for organizational purposes and understanding for readers.

Further, if I am following Table 1 correctly, the three highest ranked items supporting life-long learning are: Handling work mentally, opportunities for lifelong learning, work that can be handled physically and opportunities to get involved in research. Some of the data in Table 1 seems contrary to your explanation (for example, see statement 6). I could be misunderstanding your interpretation and if so, is there a way to construct your explanation to be clearer about the intention of those students who value lifelong learning as a calling?

Career orientation 2: Work-life balance

For consistency, begin this section the same way you began the last section – use the same structure of phrasing in your opening sentence as the previous section.

**Discussion**

The discussion section is well-organized; synthesizes the career orientation patterns found in the study; and supports salient and on-going issues broadly faced in the medical profession: attrition, work-life balance, generational differences among others. The authors also appropriately questioned the use of Q-sorting as a career guidance tool and other possible career counseling or career coaching activities. The authors identified appropriate study limitations as well as recommendations for future research.

Of particular note in the discussion section is the suggestion of intentional career development activities at the on-set as well as throughout medical school training to provide students with the opportunity for an informed perspective when it comes to making a specialty choice.

There are grammatical and punctuation errors on lines 315, 316, 372, 374
